# Genetic interaction analysis of *Candida glabrata* transcription factors *CST6* and *UPC2A* in the regulation of respiration and fluconazole susceptibility

**DOI:** 10.1128/aac.01294-24

**Published:** 2024-12-23

**Authors:** Tomye L. Ollinger, Robert Zarnowski, Josie E. Parker, Steven L. Kelly, David R. Andes, Mark A. Stamnes, Damian J. Krysan

**Affiliations:** 1 Department of Pediatrics, Carver College of Medicine, The University of Iowa 160412 , Iowa City, Iowa, USA; 2 Department of Medicine, Section of Infectious Disease, University of Wisconsin-Madison 26762 , Madison, Wisconsin, USA; 3 Department of Medical Microbiology and Immunology, University of Wisconsin-Madison 26762 , Madison, Wisconsin, USA; 4 Molecular Biosciences Division, School of Biosciences, Cardiff University 151285 , Cardiff, Wales, United Kingdom; 5 Institute of Life Sciences, Swansea University Medical School, Swansea University 7759 , Swansea, Wales, United Kingdom; 6 Department of Molecular Physiology and Biophysics, Carver College of Medicine, The University of Iowa 174490 , Iowa City, Iowa, USA; University Children's Hospital Münster, Münster, Germany

**Keywords:** *Candida glabrata*, antifungal resistance, azole, mitochondrial metabolism

## Abstract

*Candida glabrata* is the second most common cause of invasive candidiasis and is widely known to have reduced susceptibility to fluconazole relative to many other *Candida* spp. Upc2A is a transcription factor that regulates ergosterol biosynthesis gene expression under conditions of sterol stress such as azole drug treatment or hypoxia. Through an *in vitro* microevolution experiment, we found that loss-of-function mutants of the ATF/CREB transcription factor *CST6* suppresses the fluconazole hyper-susceptibility of the *upc2A*∆ mutant. Here, we confirm that the *cst6*∆ *upc2A*∆ mutants are resistant to fluconazole but not to hypoxia relative to the *upc2A*∆ mutant. Sterol analysis of these mutants indicates that this suppression phenotype is not due to restoration of ergosterol levels in the *cst6*∆ *upc2A*∆ mutant. Furthermore, increased expression of *CDR1*, the efflux pump implicated in the vast majority of azole-resistant *C. glabrata* strains, does not account for the suppression phenotype. Instead, our data suggest that this effect is due in part to increased expression of the adhesin *EPA3*, which has been shown by others to reduce fluconazole susceptibility in *C. glabrata*. In addition, we find that loss of both *UPC2A* and *CST6* reduces the expression of mitochondrial and respiratory genes and that this also contributes to the suppression phenotype as well as to the resistance of *cst6*∆ to fluconazole. These latter data further emphasize the connection between mitochondrial function and azole susceptibility.

## INTRODUCTION

Since the dawn of the anti-infective chemotherapy era, the treatment of human fungal infections has relied on a small and relatively static pharmacopeia, particularly compared with the treatment of bacterial infections and, more recently, viral infections (1). At the time of writing, three classes of antifungal drugs were used to treat life-threatening invasive fungal infections: polyenes, azoles, and echinocandins (2). Two of these drug classes, the polyenes and azoles, target fungal ergosterol homeostasis while the echinocandins inhibit the synthesis of 1,3-β-glucan, a key component of the fungal cell wall (1). The polyene and azole classes of drugs were discovered in the 1950s and 1960s, respectively. The echinocandins were initially described in the early 1970s and introduced into clinical practice in 2002; no new mechanistic classes of antifungal drugs have been U.S. Food and Drug Administration-approved in the last 22 years (1).

In addition to limiting the options available for the treatment of patients with invasive fungal infections, this small set of antifungal drugs is extremely vulnerable to the consequences of the inevitable development of antifungal drug resistance. Loss of just one class reduces the treatment options by at least 1/3 and, since the echinocandins have a relatively limited spectrum of activity, can lead to a single antifungal drug option for a critically ill patient. The most obvious solution to this problem is to develop new mechanistic classes of antifungal drugs, but the pace of this endeavor has been quite slow (see above). A second approach is to understand the fundamental mechanisms of antifungal drug resistance (3). Theoretically, such an understanding could allow the development of mechanism-based strategies to prevent or manage antifungal drug resistance.

Of the three antifungal drug classes, azole drugs have been most susceptible to the development of resistance; indeed, clinically significant azole resistance has emerged in all three of the major human fungal pathogens including *Candida*, *Aspergillus*, and *Cryptococcus* species (4). In general, azole resistance is associated with mutations affecting the expression or drug-affinity of the azole target protein, lanosterol demethylase (*ERG11* or *CYP51*), or other ergosterol biosynthesis genes or with mutations that increase the expression of plasma membrane-associated transporters presumed to be drug efflux pumps (3, 4). In the case of *Candida albicans*, azole resistance has been linked to (i) mutations causing increased *ERG11* expression (5); (ii) mutations in the Erg11 target (6); (iii) gain-of-function mutations in Upc2, a transcriptional regulator of ergosterol biosynthesis (7); and (iv) gain-of-function mutations in the transcriptional regulators of putative drug transporters (*TAC1*/*MRR1*, ref. [8]). In contrast, azole resistance in *Candida glabrata*, the second most common cause of invasive candidiasis, is almost exclusively associated with gain-of-function mutations in Pdr1, a transcription factor that, in turn, drives the expression of the ABC transporter *CDR1* (9). With that said, as antifungal drug susceptibility testing has become more widely practiced, increasing numbers of azole-resistant *C. glabrata* isolates without canonical *PDR1* mutations have been reported (10).

To identify and characterize non-*PDR1*-associated azole resistance mechanisms in *C. glabrata*, we undertook an *in vitro* microevolution approach (11). In this screen, we used a *C. glabrata* strain lacking the regulator of ergosterol biosynthesis, Upc2A, as our progenitor strain to suppress the development of *PDR1* gain-of-function mutations; deletion of *UPC2A* in a *PDR1* gain-of-function background eliminates its azole-resistant phenotype. As previously reported (11), this strategy led to the isolation of strains with loss-of-function mutations in the transcriptional repressor *ROX1* and the transcription factor *CST6*; as predicted, no *PDR1* gain-of-function mutations were isolated. Genetic and biochemical analysis of the *rox1*∆ *upc2*A∆ mutants indicated that loss of the repressor *ROX1* led to restoration of *ERG11* expression in the *upc2A*∆ mutant and inhibition of *ERG3*/*6*. These changes in ergosterol biosynthesis gene expression, in turn, led to a reduction in the ratio of ergosterol relative to the toxic sterol by-product generated by Erg11 inhibition. Therefore, the loss of *ROX1* function suppressed Upc2A azole hyper-susceptibility through direct effects on the ergosterol pathway.

Here, with the goal of understanding the mechanistic basis for the ability of a *cst6*∆ mutation to suppress the fluconazole susceptibility of the *upc2A*∆ mutant, we characterized the genetic interactions between Cst6 and Upc2A. Our analysis suggests multiple mechanisms contribute to this phenotype and highlight the role that both transcription factors play in the regulation of genes associated with mitochondrial respiration.

## RESULTS

### Deletion of *CST6* suppresses the fluconazole hyper-susceptibility of the *upc2A*∆ mutant during planktonic and biofilm growth

**FIG 1:**
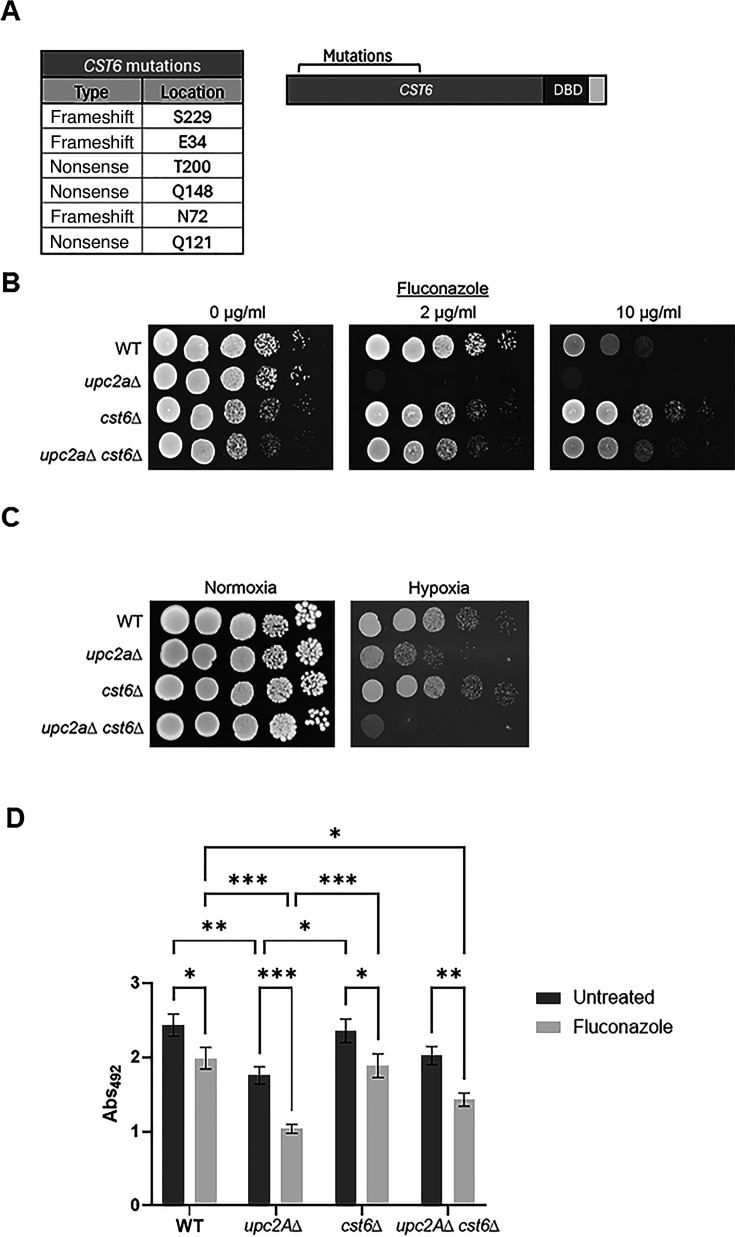
Deletion of *CST6* reduces fluconazole hyper-susceptibility of *upc2A*∆ mutants. (A) Location of *CST6* mutations in evolved strains isolated reported in reference 11 and schematic of Cst6 protein showing position relative to the DNA-binding domain (DBD). (B) A dilution series of the indicated strains were spotted on YPD and YPD with the indicated amount of fluconazole. The plates were incubated for 48 h prior to imaging. The phenotypes are representative of three biological replicates. (C) The indicated strains were plated on YPD medium and incubated in ambient air (normoxia) or in a GAS PAK (hypoxia). (D) Biofilms were generated in RPMI buffered with 0.165 M morpholino-propanesulfonic acid (MOPS) for 72 h before treatment with sham or 1000 µg/mL fluconazole. The wells were incubated for an additional 24 h, and biofilm formation was assayed by metabolic activity as described in Materials and Methods. Bars indicate mean of absorption of the XTT assay, with error bars indicating standard deviation. Asterisks indicate statistically significant differences by one-way analysis of variance followed by Tukey’s correction for multiple comparisons; *<0.05; **<0.01; ***<0.001.

As reported by Ollinger et al. (11), serial passage of *upc2A*∆ mutants in increasing concentrations led to the isolation of strains with *ROX1* and *CST6* loss-of-function mutations that were resistant to fluconazole relative to the parental strains. Of the 14 isolates, 6 contained nonsense mutations in the transcription factor *CST6* (Fig. 1A) while 6 mutants contained *ROX1* mutations. To confirm that the *CST6* loss-of-function mutations were responsible for the suppression phenotype, we constructed *cst6*∆ *upc2*A∆ double mutants and tested their susceptibility to fluconazole (Fig. 1B). As a note, we were unable to delete *CST6* in the *upc2A*∆ background but successfully constructed the strain by deletion of *UPC2A* in the *cst6*∆ background; the reason for this observation is not clear. The double mutant was, indeed, less susceptible to fluconazole relative to the *upc2A*∆ mutant and was similar to wild type (WT) at the higher fluconazole concentration. The decreased susceptibility of the *cst6*∆ mutant on spot dilution assays is consistent with our previously reported observation that its fluconazole minimum inhibitory concentration (MIC) is fourfold increased relative to WT under Clinical and Laboratory Standards Institute (CLSI) conditions (11). These data confirm that loss of *CST6* function suppresses the hyper-susceptibility of *upc2A*∆ to fluconazole and that *CST6* negatively regulates fluconazole susceptibility.

Ergosterol homeostasis is critical for *C. glabrata* growth in hypoxia as emphasized by the severe growth defect displayed by the *upc2A*∆ mutant under hypoxia (Fig. 1C). The *rox1*∆ *upc2A*∆ mutant restored ergosterol levels to WT levels in normoxia and in the presence of fluconazole. Accordingly, the *rox1*∆ mutation suppressed the inability of the *upc2A*∆ mutant to grow under hypoxic conditions (11). We, therefore, tested the growth of the *cst6*∆ *upc2A*∆ mutant in hypoxia. Somewhat surprisingly, the *cst6*∆ *upc2A*∆ mutant showed poorer growth under these conditions than the *upc2A*∆ mutant. Cst6, however, does not appear to affect hypoxic growth because the single mutant is similar to WT (Fig. 1C). These data indicate that the mechanism by which the *cst6*∆ mutation alters fluconazole homeostasis in WT and *upc2A*∆ mutants is distinct from that of the *rox1*∆ mutant.

During biofilm formation, cells at the basal layer are thought to experience hypoxia (12) and, therefore, wondered if deletion of *UPC2A* would affect the ability of *C. glabrata* to establish biofilms. The *upc2A*∆ mutant showed reduced biofilm formation (28%) as determined by the 2,3-*bis*(2-methoxy-4-nitro-5-sulfophenyl)−2H-tetrazolium-5-carboxanilide inner salt (XTT) reduction assay (Fig. 1D). Previously, Cst6 was reported to negatively regulate biofilm formation (13). We found no difference between the *cst6*∆ mutant and WT. This is most likely due to very different medium used for the two experiments since the mutants were generated in the same genetic background (BG2). Our standard conditions are RPMI + 0.25% glucose for 48 h while Riera et al. used synthetic complete medium with 2% glucose for 24 h (13). Deletion of *CST6* in the *upc2A*∆ mutant did not have a statistically significant effect on its ability to form a biofilm.

Fungal biofilms are highly resistant to fluconazole, but to our knowledge the effect of the *upc2A*∆ mutation on this phenomenon has not been previously assessed in *C. glabrata*. As shown in Fig. 1D, deletion of *UPC2A* reduced the susceptibility of the *C. glabrata* biofilm by approximately twofold while deletion of *CST6* did not have a significant effect. In contrast to the planktonic conditions, the *cst6*∆ *upc2A*∆ mutant showed no statistically significant difference in susceptibility to fluconazole relative to the *upc2A*∆ mutant. Thus, the effect of *CST6* on fluconazole susceptibility is limited to planktonic conditions.

### Loss of *CST6* function has modest effects on the ergosterol content of fluconazole treated WT or *upc2A*∆ cells and does not increase *CDR1* expression

**FIG 2:**
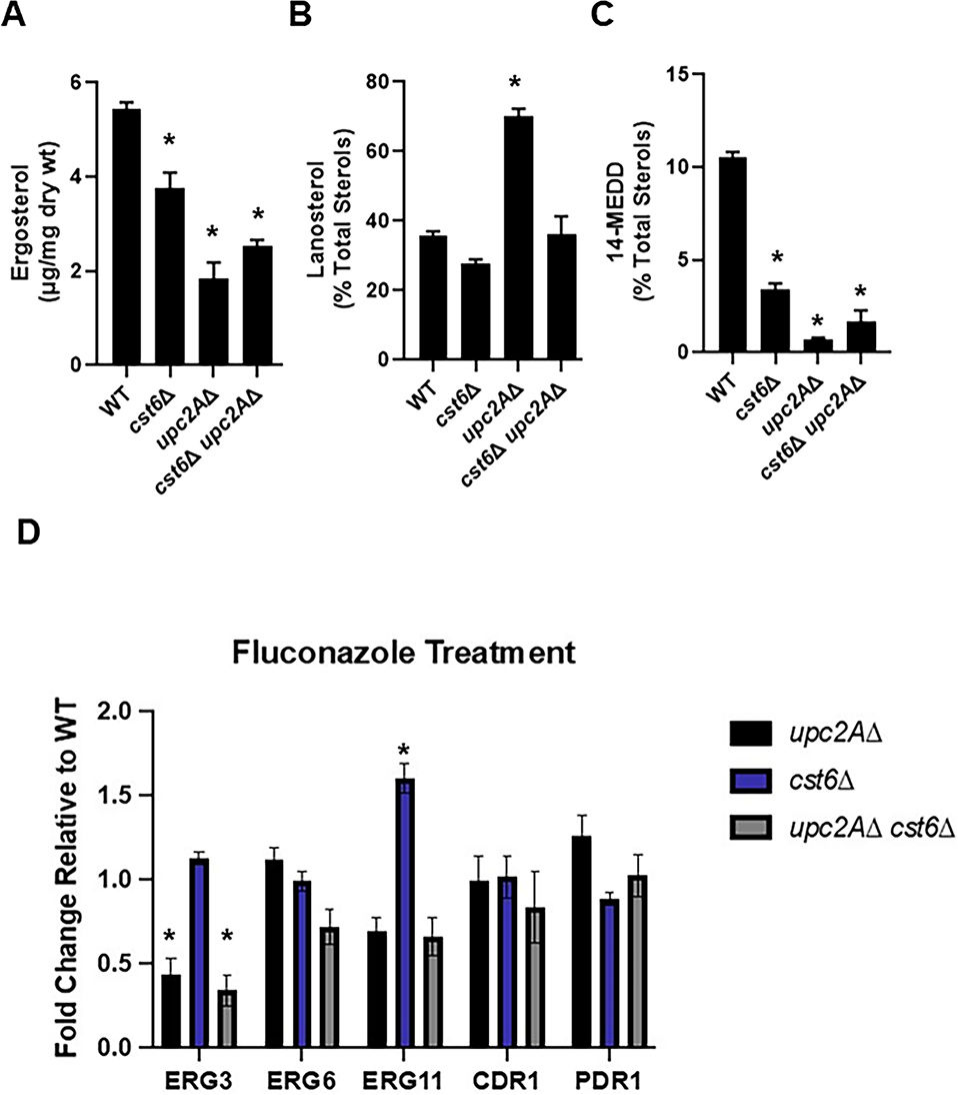
Deletion of *CST6* does not increase ergosterol levels, *ERG* gene expression or *CDR1* efflux pump expression in the presence of fluconazole. (A) The ergosterol content of the indicated strains in the presence of fluconazole (16 µg/mL) was determined as described in Materials and Methods. Bars indicate mean and error bars indicate standard deviation for three biological replicates. * indicates statistically significant difference (*P* < 0.05) from WT by one-way analysis of variance (ANOVA) and Tukey’s correction for multiple comparisons. The percentage of (B) lanosterol and (C) 14 methyl ergosta-8,24 (28)-dien-3–6-diol (14-MEDD) relative to total sterols in the indicated strains was determined as described in Materials and Methods in the presence of fluconazole (µg/mL). Full data set provided in Table S1. (D) The expression of the indicated genes relative for the *upc2*A∆, *cst6*∆, and *upc2A*∆ *cst6*∆ mutants relative to WT were determined in the presence of fluconazole (16 µg/mL) by qRT-PCR. The fold change is relative WT, and * indicates statistically significant (*P* < 0.05) difference by one-way ANOVA and Tukey’s correction for multiple comparisons. Bars indicate mean of three biological replicates performed in technical triplicate, with error bars indicating standard deviation.

One explanation for the distinct hypoxia phenotypes shown between the *cst6*∆ *upc2A*∆ and *rox1*∆ *upc2A*∆ mutants is that the *cst6*∆ mutation may not reduce the fluconazole susceptibility of the *upc2A*∆ mutant by increasing the ergosterol content of the double mutant as the *rox1*∆ mutation does. To test this hypothesis, we determined ergosterol content and characterized the distribution of sterols in the WT, *cst6*∆, *upc2A*∆, and *cst6*∆ *upc2A*∆ mutants in the presence of fluconazole (Table S1). As expected, the ergosterol content of the *upc2A*∆ mutant is dramatically reduced relative to WT in fluconazole; ergosterol content of the *cst6*∆ mutant is also reduced but not the extent of the *upc2A*∆ mutant (Fig. 2A). The *cst6*∆ *upc2A*∆ double mutant has slightly higher ergosterol levels compared with the *upc2A*∆ mutant but those levels are still twofold lower than WT. Thus, an increase in ergosterol content may contribute to the fluconazole resistance of the *cst6*∆ *upc2A*∆ mutant relative to the *upc2A*∆ mutant, but it seems unlikely that this is the sole mechanism.

Inhibition of Erg11 leads to a build-up in lanosterol and the accumulation of a toxic sterol (14 methyl ergosta-8,24 (28)-dien-3–6-diol [14-MEDD] [14]). Fluconazole treatment of WT cells increases the percentage of lanosterol by approximately 10-fold relative to untreated cells (11). In the *upc2A*∆ mutant, lanosterol levels are twofold higher than WT but lanosterol levels are similar to WT for the *cst6*∆ and *cst6*∆ *upc2A*∆ mutants. Therefore, loss of *CST6* blunts the lanosterol accumulation observed for the *upc2A*∆ mutant in the presence of fluconazole. However, the proportions of the toxic sterol 14-MEDD are reduced in all three mutants (*cst6*∆, *upc2*A∆, and *cst6*∆ *upc2A*∆ mutants) relative to WT (Fig. 2C). Therefore, altered levels of 14-MEDD do not explain the suppression of *upc2A*∆ fluconazole hyper-susceptibility by deletion of *CST6*. These data indicate that loss of Cst6 leads to reduced fluconazole susceptibility through mechanisms that appear to be largely unrelated to changes in sterol homeostasis.

Next, we asked if loss of Cst6 function affected expression of genes with well-characterized effects on fluconazole susceptibility. Increased expression of the fluconazole target *ERG11* would be expected to reduce susceptibility as has been described previously (5). Also, loss-of-function mutants of *ERG3* and *ERG6* reduce fluconazole susceptibility in *C. glabrata*. As previously reported (11), the *upc2A*∆ mutant has reduced expression of *ERG3* and *ERG11* relative to wild type in the presence of fluconazole (16 µg/mL) while the *cst6*∆ mutant had modestly increased expression of *ERG11* (Fig. 2D). The *cst6*∆ *upc2A*∆ mutant, however, showed *ERG* gene expression levels that were essentially unchanged from the *upc2A*∆ mutant. This indicates that changes in *ERG* gene expression cannot account for the reduced fluconazole susceptibility of the *cst6*∆ *upc2A*∆ mutant relative to the *upc2A*∆ mutant. However, the twofold reduction in *ERG11* expression in the *cst6*∆ *upc2A*∆ mutant relative to the *cst6*∆ mutant suggests that the modest increase in *ERG11* expression in the *cst6*∆ mutant is Upc2A-dependent.

The most common mechanism of acquired fluconazole resistance in *C. glabrata* is gain-of-function mutations in the transcription factor Pdr1, leading to increased expression of the putative efflux pump *CDR1* (15). We, therefore, asked if loss of *CST6* function led to altered expression of either *PDR1* or *CDR1*. However, neither *PDR1* nor *CDR1* expression is significantly different from WT in any of the three mutants (Fig. 2D). Taken together, these data indicate that the reduced fluconazole susceptibility of the *cst6*∆ mutant and the *cst6*∆ *upc2A*∆ mutant is not due to altered ergosterol content, sterol distribution, or expression of efflux pumps.

### Cst6 is a transcriptional activator and repressor that regulates cell wall adhesin, ergosterol homeostasis, and mitochondrial and carbon metabolism genes

Next, we carried out RNA-seq-based profiling of the single mutants, double mutants, and WT strains in the presence and absence of fluconazole. The *upc2A*∆ mutant has been characterized previously, and our overall results were similar. The effect of the *CST6* deletion on the genome-wide transcriptional profile of *C. glabrata* has not been reported while focused studies have identified genes that are modulated by Cst6. Under biofilm conditions, the cell wall adhesion gene *EPA6* is upregulated approximately twofold in the *cst6*∆ mutant. Cst6 has also been shown to positively regulate *NCE103,* which codes for carbonic anhydrase (16).

**FIG 3:**
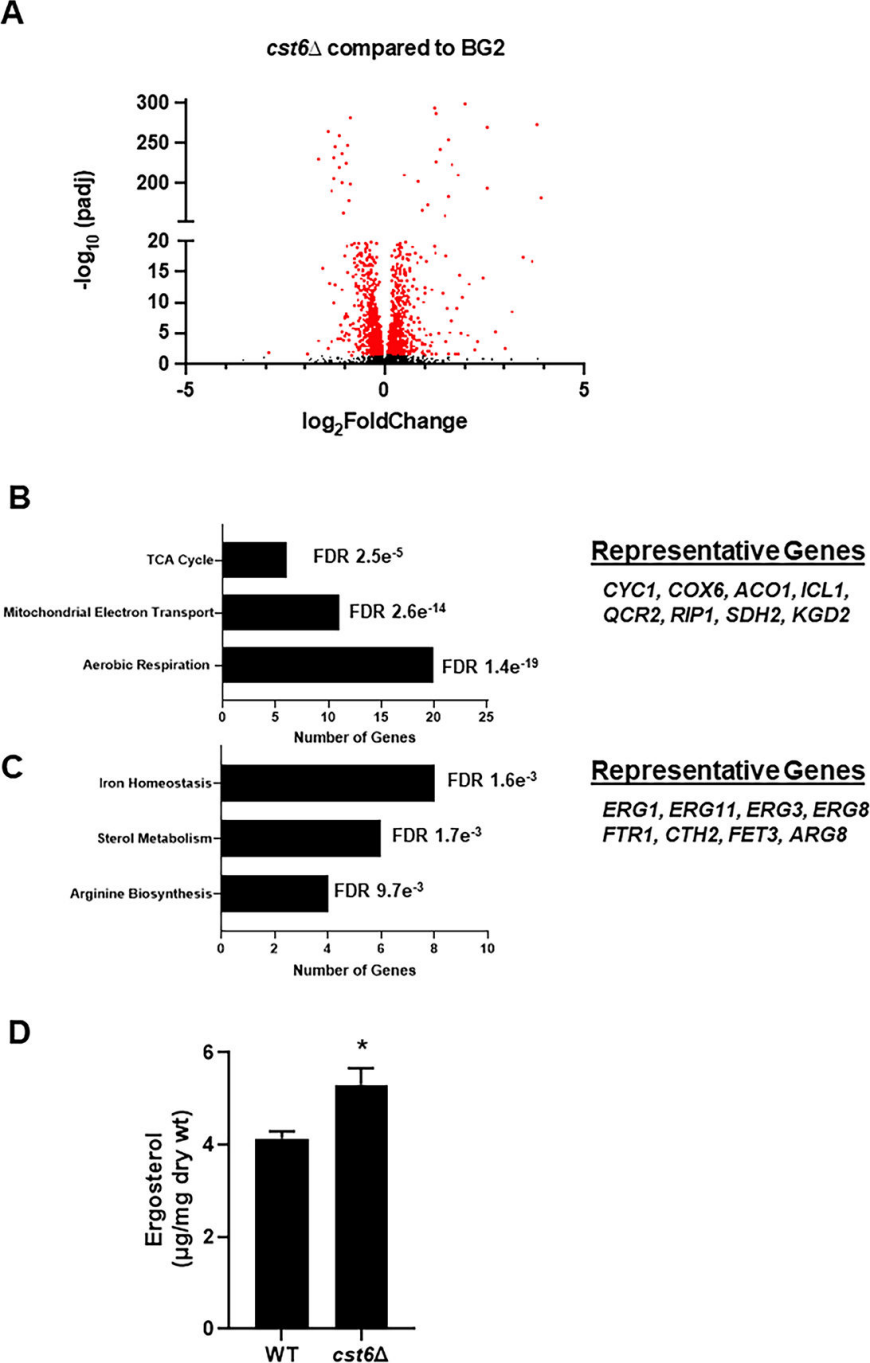
Cst6 is both a positive and negative regulator of gene expression. (A) Volcano plot of RNA-seq data comparing the *cst6*∆ mutant to the BG2 reference strain. Red dots indicate differentially expressed genes (log_2_ ±1 and false discovery rate [padj] <0.05) and black dots are genes whose expression does not change significantly. Biological process GO terms enriched in the set of genes downregulated (B) and upregulated (C) in the *cst6*∆ mutant, with the number of genes in each GO term group listed on the x-axis. The FDR was determined by Benjamini-Hochberg analysis. (D) The ergosterol content of BG2 and *cst6*∆ during logarithmic phase growth in YPD. Bars indicate mean of three biological replicates, with error bars indicating standard deviation. * indicates *P* < 0.05 by Student’s *t* test.

**FIG 4:**
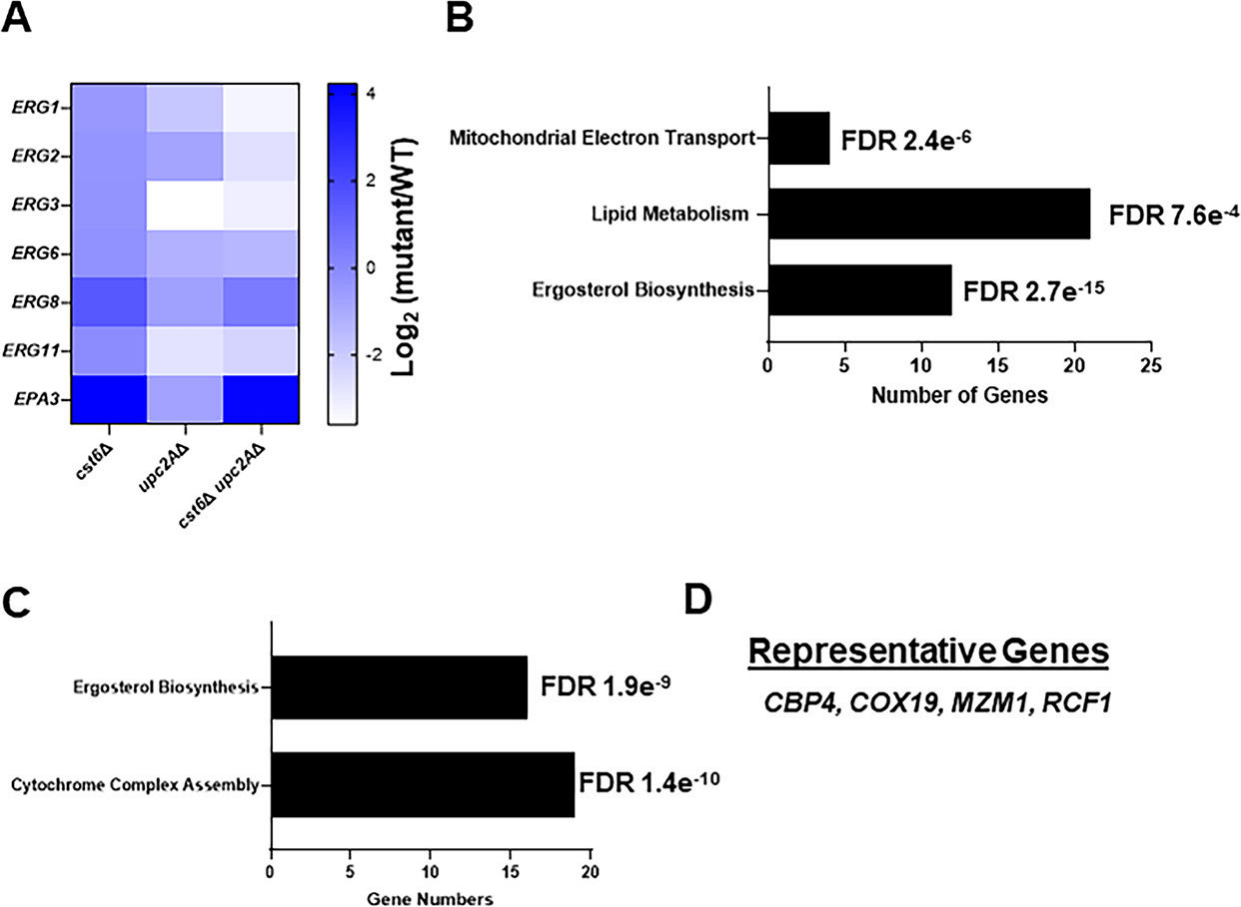
Effect of the *cst6*∆ *upc2A*∆ mutant on gene expression relative to single mutants in the presence of fluconazole. (A) Heatmap comparing the expression (RNA-seq) of the indicated *ERG* genes and the adhesin *EPA3* for the *upc2*A∆, *cst6*∆, and *upc2A*∆ *cst6*∆ mutants relative to WT (BG2). Biological process GO term analysis of genes downregulated in the *upc2A*∆ *cst6*∆ (B) and *upc2A*∆ (C) mutants in the presence of fluconazole with the number of genes in each GO term group listed on the x-axis. The FDR was determined by Benjamini-Hochberg analysis. (D) Representative mitochondrial and respiration genes downregulated in the *upc2A*∆ mutant is shown.

We characterized the expression profile of the *cst6*∆ mutant in the absence and presence of fluconazole. In yeast, peptone, and dextrose (YPD) at 30°C, 66 genes were downregulated and 115 were upregulated (differentially expressed gene defined as log_2_ ±1 relative to WT; false discovery rate [FDR] < 0.05; Fig. 3A; Table S2). Gene Ontology (GO) term analysis (Fig. 3B) of the set of downregulated genes showed that it was enriched for genes involved in aerobic respiration (1.4e^−19^) and mitochondrial electron transport (1.6e^−15^). Consistent with previous reports (16), *NCE103* expression was reduced (−1 log_2_, FDR < 0.0001). By biological process GO term analysis, iron homeostasis, ergosterol biosynthesis, and arginine biosynthesis genes were the most enriched functional groups for the set of genes upregulated genes in the *cst6*∆ mutant (Fig. 4B). As previously reported, *EPA6* was upregulated (3.4 log_2_, FDR < 1e^−100^) as were two other *EPA* family adhesins (*EPA2*, *EPA3*). Indeed, the top cellular component GO term for the upregulated genes was cell wall (nine genes, FDR 0.01).

Because four *ERG* genes including *ERG11* were upregulated in the *cst6*∆ mutant, we determined the ergosterol content of the *cst6*∆ mutant during log phase growth in YPD at 30°C. Under these conditions, the ergosterol content of the *cst6*∆ mutant is increased by 20% relative to WT (Fig. 3D). Although *ERG* gene expression and ergosterol content are not increased in the fluconazole-treated *cst6*∆ mutant, it seems possible that the increased baseline expression of these genes and ergosterol content of the cell may partially contribute to the fluconazole resistance of the *cst6*∆ mutant.

In the presence of fluconazole, 18 genes were downregulated in the *cst6*∆ mutant and 143 are upregulated relative to WT (Table S2). No specific class of genes was enriched in the set of genes that was downregulated. The set of upregulated genes in the fluconazole-treated *cst6*∆ mutant was enriched for ribosome biogenesis (FDR = 0.0004) and cell wall (FDR = 0.05) genes. *EPA3* was the adhesin for which expression was increased the most (log_2_ 4.2; FDR = 3e^−219^). Recently, increased expression of the adhesin *EPA3* was found to increase fluconazole resistance in WT strains (17). Indeed, strains with increased expression of *EPA3* were recovered from an *in vitro* evolution experiment in the presence of azole drug. Accordingly, the increased expression of *EPA3* could contribute to the decreased fluconazole susceptibility of the *cst6*∆ mutant. Of the four *ERG* genes upregulated in the untreated *cst6*∆ mutant, only *ERG8* expression was increased relative to WT in the presence of fluconazole (log_2_ 1.6; FDR = 0.0006). Therefore, it is not clear that the increased expression of *ERG* genes makes a significant contribution to the resistance of the *cst6*∆ mutant to fluconazole. Finally, Cst6 clearly functions as both a suppressor and activator of gene expression in *C. glabrata* based on the large number of genes that have increased expression in the deletion mutant.

### Deletion of *CST6* in the *upc2A*∆ mutant increases adhesin gene expression and reduces respiratory gene expression

To identify genes whose differential expression may be related to the ability of *cst6*∆ to suppress *upc2A*∆ fluconazole susceptibility, we compared the expression profiles of the *upc2A*∆ mutant to the *cst6*∆ *upc2A*∆ double mutant in the presence of fluconazole (Table S2). The expression profile of the *upc2A*∆ mutant in the presence of fluconazole has previously been reported by us and others. As expected from these data and the single-gene expression data reported above, the expression of ergosterol biosynthesis genes was reduced significantly in the *upc2A*∆ mutant but was not restored in *cst6*∆ *upc2A*∆ double mutant (Fig. 4A). The only ergosterol biosynthesis-related gene with increased expression in the double mutant relative to *upc2A*∆ was *ERG8. ERG8* expression does not appear to be regulated by Upc2A during fluconazole exposure because its expression is not significantly changed in the *upc2A*∆ mutant relative to WT (Table S1). These data firmly establish that deletion of *CST6* does not suppress the fluconazole hypersensitivity of the *upc2A*∆ mutant by modulation of *ERG* gene expression.

The elevated expression of adhesin *EPA3* observed in the *cst6*∆ mutant is maintained in the *cst6*∆ *upc2A*∆ double mutant with *EPA3* expression increased 16-fold relative to both the *upc2A*∆ and WT strains (Fig. 4A). Thus, elevated expression of *EPA3* is a potential mechanism for the suppressive effect of the *cst6*∆ mutation on *upc2A*∆ fluconazole hyper-susceptibility. We attempted to delete *EPA3* in the *cst6*∆ and the *cst6*∆ *upc2A*∆ double mutant to determine if loss of *EPA3* would increase the fluconazole susceptibility of those strains. We were, however, unable to generate *epa3*∆ mutants in the *cst6*∆ and the *cst6*∆ *upc2A*∆ double mutant. Similarly, we were unable to clone *EPA3* into an overexpression cassette to determine if increased *EPA3* expression would suppress *upc2A*∆ fluconazole hyper-susceptibility. We suspect that these technical difficulties are related to three factors: (i) the highly repetitive sequences of *EPA*3, (ii) the closely related sequences of the *EPA* family members and (iii) their presence in the sub-telomeric regions of the chromosomes.

The most downregulated gene in the *cst6*∆ *upc2A*∆ double mutant relative to WT is subunit 1 of the cytochrome *c* oxidase (*COX1,* log_2_ −22.6, FDR 5.3e^−17^). Similarly, *COX2* (log_2_ −5.25, FDR 0.05) and *COX3* (log_2_ −6.28, FDR 0.04) are downregulated significantly in the *cst6*∆ *upc2A*∆ double mutant. GO term analysis indicates that the set of downregulated genes in the *cst6*∆ *upc2A*∆ double mutant is enriched for ergosterol biosynthesis, lipid metabolism, and mitochondrial electron transport (Fig. 4B). *COX1*, *COX2*, and *COX3* are encoded in the mitochondrial genome. Two additional mitochondrially encoded genes CaglfMr13 (log_2_ −2.45, FDR 0.019) and CaglfMr14 (log_2_ −2.59, FDR 0.0079) are also significantly downregulated in the *cst6*∆ *upc2A*∆ double mutant.

Upc2A has not previously been associated with the regulation of mitochondrial or respiratory metabolic genes. We, therefore, examined the expression of these genes in our *upc2A*∆ mutant profile. The two top GO terms for the set of genes downregulated in the *upc2A*∆ mutant in fluconazole were cytochrome complex assembly and ergosterol biosynthesis (Fig. 4C and D). Interestingly, Cst6 affected the expression of mitochondrially encoded genes while Upc2A affected the expression of cytochrome assembly genes encoded in the nuclear chromosomes. In the absence of fluconazole, Upc2A only affects the expression of nine genes, none of which are involved in ergosterol biosynthesis or respiration (Table S2). Therefore, it appears that both Upc2A and Cst6 play a role in the expression of respiratory genes but do so by regulating distinct sets of electron transport genes.

### Forced respiration increases the susceptibility of the *upc2A*∆ mutant to fluconazole

Reduced mitochondrial function has been linked to reduced fluconazole susceptibility by many previous studies (18, 19). Most dramatically, loss of mitochondrial DNA leading to *rho*^0^, petite cells led to increased expression of the ABC transporter *CDR1* through the activation of the transcription factor Pdr1. Petite and *rho*^0^ cells such as these are unable to grow on non-fermentable but are highly resistant to fluconazole when grown on glucose (19). Indeed, we isolated multiple petite strains from the original *in vitro* microevolution experiment with the upc2A∆ mutant (11). Kaur et al. isolated fluconazole-resistant transposon insertion mutants in mitochondrial genes that were not formally petite (retained mitochondrial genome) but were functionally petite in a reversible manner (18). These data suggest that reduced respiratory capacity but not complete loss of mitochondrial function may be sufficient to alter fluconazole susceptibility.

**FIG 5:**
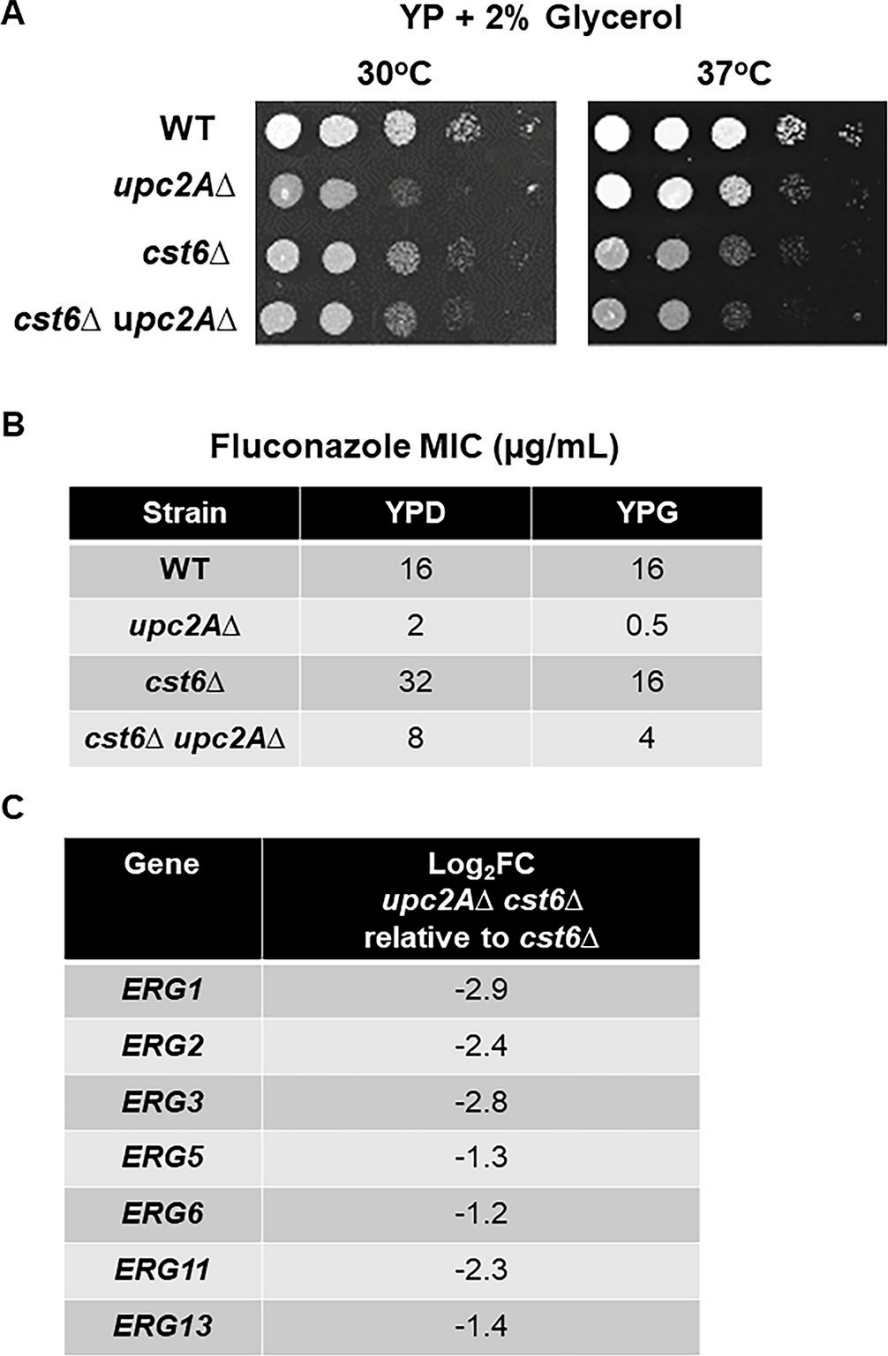
Forced respiration with glycerol medium increases fluconazole susceptibility of *cst6*∆, *upc2A*∆, and *upc2A*∆ *cst6*∆ mutants. (A) The *upc2*A∆, *cst6*∆, and *upc2A*∆ *cst6*∆ mutants were plated on YP + 2% glucose and YP + 2% glycerol medium and incubated at 30°C or 37°C for 48–72 h. (B) The minimum inhibitory concentration (MIC) of fluconazole was determined after incubation for 24 h (glucose) or 48 h (glycerol) at 37°C. (C) The effect of loss of Upc2A function on the expression of ERG genes in the *cst6*∆ mutant by RNA-seq. Full data set is in Table S2. All changes were statistically significant (adjusted *P* < 0.05).

Neither *cst6*∆ nor *upc2A*∆ mutants have been reported to show petite phenotypes. However, their expression profiles strongly suggested that the mutants may have reduced respiratory activity. Yeast that are cultivated in non-fermentable carbon sources such as glycerol are completely dependent upon respiration. We, therefore, tested the growth of these mutants on glycerol medium (YP + 2% glycerol) to determine if they have reduced fitness when forced to respire. At 24 h, the *upc2A*∆, *cst6*∆, and *cst6*∆ *upc2A*∆ mutants all showed reduced growth relative to WT at both 30°C and 37°C (Fig. 5A). Although this phenotype is modest, it was consistent across three independent isolates of the *upc2A*∆ *cst6*∆ mutant (Fig. S1A). We hypothesized that the rich nature of YP medium may modulate the glycerol effect by providing glucogenic amino acids. Therefore, we examined the growth of WT and the four mutants on YNB medium with 2% glucose or glycerol. Surprisingly, the *upc2A*∆ *cst6*∆ mutant had very poor growth on YNB with either carbon source (Fig. S1B), making it impossible to assess the effect of glycerol on the growth of the double mutant. The *cst6*∆ and *upc2A*∆ mutants grew similarly to WT on YNB + 2% glucose while the *cst6*∆ mutant had a much stronger growth phenotype on YNB + 2% glycerol than on YP + 2% glycerol (Fig. S1B); the *upc2A*∆ mutant had a minimal phenotype on YNB + 2% glycerol. Taken together, these observations support the conclusion that strains containing *cst6*∆ mutations have reduced respiratory capacity.

These data are consistent with the conclusion that the mutants have reduced but not absent respiratory activity. Indeed, this reduction in respiratory activity is not profound and is certainly not to the extent observed for petite or *rho*^0^ strains (19). To our knowledge, decreased respiratory fitness has not previously been observed for the *upc2A*∆ mutant in *C. glabrata* or for *UPC2* mutants in other species. Mutants of *S. cerevisiae CST6*, however, have been reported to show reduced growth on non-glucose carbon sources (20). Taken together, the glycerol phenotypes for these mutants are consistent with the observed reduction in the expression of respiratory and mitochondrial electron transport genes.

Next, we asked if reduced respiratory activity contributed suppression of the increased fluconazole susceptibility of *upc2A*∆ mutant by deletion of *CST6*. To test this hypothesis, we determined the MIC of fluconazole in medium with glycerol as the primary carbohydrate carbon source. The MIC of fluconazole toward WT was the same in YPD and yeast, peptone, and 2% glycerol (YPG) (Fig. 5B). The fluconazole MIC was reduced fourfold and twofold for the *upc2A*∆ and *cst6*∆ mutants, respectively (Fig. 5B). These data indicate that the reduced expression of mitochondrial and respiratory genes and the resulting reduced respiratory capacity of strains lacking *UPC2A* and *CST6* affects the strains susceptibility to fluconazole. The fluconazole MIC of the *cst6*∆ *upc2A*∆ mutant is also reduced twofold in glycerol medium relative to glucose medium but remains eightfold above the MIC of the *upc2A*∆ mutant (Fig. 5B). Consequently, the *cst6*∆ mutation suppresses the fluconazole hyper-susceptibility through a mechanism that is only partially dependent on the altered expression of genes associated with respiration.

Finally, in both glucose and glycerol, deletion of *UPC2A* increases the susceptibility of the *cst6*∆ mutant. We, therefore, directly compared the expression of ERG genes in the RNA-seq data sets for the *cst6*∆ mutant and the *cst6*∆ *upc2A*∆ mutant in the presence of fluconazole. Indeed, the expression of seven ERG genes was significantly reduced in the *cst6*∆ *upc2A*∆ mutant relative to the *cst6*∆ mutant (Fig. 5C). These observations indicate that the reduced susceptibility of the *cst6*∆ mutant relative to WT is partially dependent upon Upc2A. This is consistent with previous reports from the Rogers lab indicating that loss of Upc2A function increases the fluconazole susceptibility of mutants that are resistant to fluconazole due to multiple mechanisms (21).

## DISCUSSION

*C. glabrata* Upc2A, along with homologs in other pathogenic yeast, regulates the expression of ergosterol biosynthesis genes (21). The preponderance of evidence from multiple organisms indicates that Upc2A is localized to the cytosol in an inactive state when ergosterol levels are in homeostasis. Upon reduction in ergosterol levels under hypoxic conditions or in the presence of ergosterol biosynthesis inhibitors such as azole drug, Upc2A is trafficked from the cytosol to the nucleus where it promotes the expression of ergosterol biosynthesis genes. Recent structural studies suggest that Upc2 orthologs may directly bind ergosterol as part of a sensing function (22). Upc2A and its homologs, therefore, seem to regulate ergosterol biosynthesis only during sterol stress.

Gain-of-function *UPC2* mutants in *C. albicans* (7) and *S. cerevisiae* (23) cause reduced susceptibility to azole drugs. On the other hand, deletion of *UPC2A* overcomes the fluconazole resistance caused by increased expression of the ABC transporter *CDR1* (21) However, no fluconazole-resistant *C. glabrata* clinical isolates with *UPCA2* mutations have been identified. Indeed, the vast majority of *C. glabrata* clinical isolates have increased *CDR1* expression. Here, we demonstrate that deletion of the transcription factor Cst6 suppresses the fluconazole susceptibility of the *upc2A*∆ mutant and decreases susceptibility of WT strains to fluconazole. Characterization of the genetic interaction between *CST6* and *UPC2A* has provided new insights into the function of both transcription factors and the potential mechanisms of the suppression phenotype.

First, we show that Cst6 modulates the expression of a large number of genes, both positively and negatively. Like its homologs in other yeast, it has been previously shown to regulate the expression of carbonic anhydrase (*NCE103*); however, it cannot be the sole regulator of this gene because the *cst6*∆ mutant can grow in low CO_2_ conditions, whereas the *nce103*∆ cannot (16). The *C. glabrata cst6*∆ mutant has also been linked to repression of biofilm formation that has been further associated with increased expression of the adhesin *EPA6* (13). We confirmed that *EPA6* expression is increased in the *cst6*∆ mutant and found that other adhesins and cell wall proteins were also upregulated. We also found that *ERG* gene expression was increased while genes related to mitochondrial respiration were downregulated. The latter observation, along with the reduced fitness of the *cst6*∆ mutant on nonfermentable carbon sources such as glycerol, is consistent with the phenotypes reported for *S. cerevisiae* homologs of Cst6 (20). Thus, *C. glabrata* Cst6 has an extensive regulon, functions as both an activator and a repressor of gene expression, and plays important roles in the regulation of cell wall and carbon metabolism gene expression.

Second, the mechanistic basis for the ability of the *cst6*∆ mutation to suppress the fluconazole hyper-susceptibility of the *upc2A*∆ mutant appears to be multifactorial and related to at least two effects that the *cst6*∆ mutation has on gene expression. Consistent with previous observations (13), he *cst6*∆ mutation increases the expression of adhesin genes including *EPA3*. Increased expression of *EPA3* has been linked to fluconazole resistance through both *in vitro* evolution experiments and through genetic analysis (17). The strong upregulation of *EPA3* in the *cst6*∆ *upc2A*∆ mutant is, therefore, likely to contribute to its relative fluconazole resistance compared with the single *upc2A*∆ mutant.

Loss of Cst6 and, to a lesser extent, Upc2A directly or indirectly reduces the expression of mitochondrial and respiration-associated genes. Consistent with this transcriptional effect, the *upc2A*∆ and *cst6*∆ mutants have reduced respiratory capacity based on modest growth defects in glycerol medium (Fig. 5A). Reduced respiration is associated with decreased susceptibility to fluconazole (18, 19). Although complete loss of mitochondrial function is associated with the highest levels of fluconazole resistance (18), transient reductions in mitochondrial function have also been described to reduce fluconazole susceptibility (19, 24). The fluconazole MIC is reduced in glycerol relative to glucose for the *upc2A*∆, *cst6*∆, and *cst6*∆ *upc2A*∆ mutants. Therefore, we suggest that the reduced respiratory capacity of the cells in glucose contributes to the fluconazole susceptibility of those strains under those conditions.

In the case of the *upc2A*∆ mutant, reduced respiratory capacity appears to buffer the effects of reduced ERG gene expression in the presence of glucose. It is important to emphasize that ERG gene expression is not zero in the *upc2A*∆ mutant. As such, the reduced expression of respiratory-associated genes in the *upc2A*∆ mutant seems to represent a compensatory response that allows growth under conditions of low ergosterol biosynthesis. For the *cst6*∆ *upc2A*∆ mutant, our data support the conclusion that increased expression of *EPA3,* along with reduced expression of respiratory genes, contributes to the ability of the *cst6*∆ mutation to suppress the fluconazole hyper-susceptibility of the *upc2A*∆ mutant.

Third, our work on Cst6 is consistent with a growing body of literature indicating that homologs of this ATF/CREB transcription factor play a general role in the regulation of fluconazole susceptibility. First, the Sanglard lab has reported that deletion of *RCA1*, the *C. albicans* homolog of Cst6, reduces fluconazole susceptibility (24). Interestingly, Vandeputte et al. found that deletion of *RCA1* in *C. albicans* reduced ergosterol content relative to the parental strain while we observed that deletion of *CST6* in *C. glabrata* increased ergosterol content (Fig. 3D). Although this may represent a species-specific rewiring, we measured ergosterol in logarithmic phase cells and they measured levels with stationary phase cells (24). Second, the Cunningham lab found that transposon insertions in *C. glabrata CST6* increased fitness in a Tn-seq experiment under fluconazole selection (25). Third, a genome-wide association study identified two SNPs in the promoter of *CST6* that were associated with fluconazole susceptibility; however, this was a small study and additional confirmatory work is needed to confirm this association.

Although our studies have not provided a definitive single mechanism by which loss of *CST6*/*RCA1* function leads to decreased susceptibility to fluconazole, we have identified three mechanisms that are likely to contribute to this phenotype: (i) increased baseline *ERG* gene expression and ergosterol content in logarithmic phase; (ii) increased expression of *EPA3*; and (iii) reduced expression of mitochondria/respiratory gene expression. As more clinical isolates of fluconazole-resistant *C. glabrata* are studied, it will be interesting to see if mutations in *CST6* may contribute to Pdr1-Cdr1-independent fluconazole resistance.

## MATERIALS AND METHODS

### Strains, media, and chemicals

All strains were generated in the BG2 *C. glabrata* genetic background. The *cst6*∆ and *upc2A*∆ mutants have been reported previously (11). The *cst6*∆ *upc2A*∆ mutant was constructed by sequential deletion of the two open reading frames (ORFs) using nourseothricin and hygromycin markers using the transformation method described by Istel et al. (26). Genotypes were confirmed by PCR analysis of the integration sites and lack of products with primers for the regions deleted. The primers used for these manipulations are provided in Table S3. Yeast peptone dextrose and glycerol media were prepared using standard recipes (27). Strains were pre-cultured overnight in YPD at 30°C with shaking prior to use in all subsequent assays. Fluconazole was obtained from Sigma-Aldrich.

### Spot dilution assays

Cultures were grown overnight in liquid YPD at 30°C at 200 rpm. One milliliter of culture was spun down and rinsed twice with phosphate-buffered saline (PBS). Cells were diluted to (OD) of 1 and plated with 10-fold serial dilutions on their respective media YPD, YPD with 2 µg/mL or 10 µg/mL fluconazole, or YPG. Plates were incubated at 30°C or 37°C. For the hypoxia experiments, plates were sealed in BD GasPak EZ Anaerobe Gas Generating Pouch System in a 30^o^C incubator. Images of all plates were captured after 48 h.

### Sterol analysis

Overnight cultures from single colonies of *C. glabrata* strains were used to inoculate 20 mL YPD (starting OD_600nm_ 0.20) in the absence (dimethylsulfoxide [DMSO] control, 1% v/v) or presence of 16 µg/mL fluconazole (stock prepared in DMSO, final concentration 1% v/v DMSO). Cultures were grown at 30°C for 16 h at 180 rpm. Cells were then pelleted and washed with ddH_2_O before splitting each sample for sterol extraction and dry weight determination. Sterols were extracted and derivatized as previously described (28). An internal standard of 5 µg of cholesterol was added to each sample, and lipids were saponified using alcoholic KOH and non-saponifiable lipids extracted with hexane. Samples were dried in a vacuum centrifuge and were derivatized by the addition of 0.1 mL BSTFA TMCS (99:1, Sigma) and 0.3 mL anhydrous pyridine (Sigma) and heating at 80°C for 2 h. TMS-derivatized sterols were analyzed and identified using GC/MS (Thermo 1300 GC coupled to a Thermo ISQ mass spectrometer, Thermo Scientific) and Xcalibur software (Thermo Scientific). The retention times and fragmentation spectra for known standards were used to identify sterols. Integrated peak areas were determined to calculate the percentage of total sterols. Ergosterol quantities were determined using standard curves of peak areas of known quantities of cholesterol and ergosterol. Sterol composition and ergosterol quantities were calculated as the mean of three replicates. The statistical significance of the differences between strains was determined using the means and standard error of the means and Student’s *t* test with *P* < 0.05 indicating statistical significance.

### MIC determination

All strains were cultured overnight in YPD at 30°C. One milliliter of each culture was spun down and washed twice with sterile PBS. Twofold dilution series were prepared for fluconazole in YPD and YPG, and 1 × 10^3^ cells were added to each well. Plates were incubated at 37°C for 24 h.

### Biofilm growth and fluconazole susceptibility determination assay

The growth and susceptibility of *C. glabrata* biofilms to fluconazole were assessed in 96-well flat-bottom polystyrene plates. Fluconazole was used at a concentration of 1,000 mg/mL. Fungal cell inocula (10⁶ cells/mL) were prepared from overnight yeast cultures in YPD at 30°C, then diluted in RPMI-MOPS based on cell counts obtained with an automated Countess II cell counter (Invitrogen). Each well was seeded with 100 µL of yeast cells and incubated for 24 h at 37°C to allow biofilm formation. The biofilms were gently washed with PBS (pH 7.2) to remove nonadherent cells, followed by treatment with a single dose of fluconazole. Nontreated control wells received an equal volume of saline. After an additional 24 h incubation, biofilm growth dynamics and susceptibility to fluconazole were evaluated using the colorimetric XTT reduction assay. Fresh XTT was prepared at 0.75 mg/mL, and 1 mM menadione was added to enhance XTT reduction. Absorbance at 492 nm was measured using an automated Cytation 5 imaging reader (BioTek). The percent reduction in biofilm growth was calculated by comparing the absorbance of treated wells to that of the untreated controls.

### Isolation of RNA and qRT-PCR

Cells were grown overnight in liquid YPD at 30°C at 200 rpm, back diluted into fresh YPD, and grown for 4 h. Cultures were split at mid-log phase, with one sample treated with 16 µg/mL of fluconazole and the other representing a no-drug control. Cultures were incubated for 4 h and then harvested. MasterPure Yeast RNA Purification Kit was used to isolate total RNA, which was used for qRT-PCR and for RNA-seq as described below. For qRT-PCR, iScript cDNA synthesis kit (170-8891; Bio-Rad) was used for reverse transcription. IQ SyberGreen Supermix (170-8882; Bio-Rad) was used for qPCR and primers reported in Table S3.

### RNA-seq methods and analysis

RNA samples were quantified using Qubit 2.0 Fluorometer (Life Technologies, Carlsbad, CA, USA), and RNA integrity was checked using Agilent TapeStation 4200 (Agilent Technologies, Palo Alto, CA, USA). The RNA sequencing libraries were prepared using the NEBNext Ultra II RNA Library Prep Kit for Illumina using manufacturer’s instructions (New England Biolabs, Ipswich, MA, USA). Briefly, mRNAs were initially enriched with Oligod(T) beads. Enriched mRNAs were fragmented for 15 min at 94°C. First-strand and second-strand cDNA were subsequently synthesized. cDNA fragments were end repaired and adenylated at 3′ ends, and universal adapters were ligated to cDNA fragments, followed by index addition and library enrichment by PCR with limited cycles. The sequencing libraries were validated on the Agilent TapeStation (Agilent Technologies, Palo Alto, CA, USA) and quantified by using Qubit 2.0 Fluorometer (Thermo Fisher Scientific, Waltham, MA, USA), as well as by quantitative PCR (KAPA Biosystems, Wilmington, MA, USA). The sequencing libraries were clustered on one flowcell lane. After clustering, the flowcell was loaded on the Illumina HiSeq instrument (4000 or equivalent) according to the manufacturer’s instructions. The samples were sequenced using a 2 × 150 bp paired end configuration. Image analysis and base calling were conducted using the Control software. Raw sequence data (.bcl files) generated from the sequencer were converted into fastq files and de-multiplexed using Illumina’s bcl2fastq 2.17 software. One mismatch was allowed for index sequence identification.

The quality of read files was confirmed using FastQC (Babraham Institute). Read files were mapped to *C. glabrata* CBS138 reference genome v62 (FungiDB) using HISAT2, and gene counts were obtained using Stringtie (29). Differential expression fold change, Wald test *P* values, and Benjamini-Hochberg adjustment for multiple comparisons were determined using DESeq2 (30). The absence of batch effects was confirmed using principal component analysis on regularized log-transformed gene counts. The RNA-seq data sets are provided in Table S2 and are deposited at the GEO Omnibus site.

## SUPPLEMENTAL MATERIAL

Fig. S1 aac.01294-24-s0001.tif Additional phenotypes on glycerol-containing medium.

Supplemental legends aac.01294-24-s0002.docx Legends for supplemental tables and figure.

Table S1 aac.01294-24-s0003.xlsx Sterol profiles.

Table S2 aac.01294-24-s0004.xlsx RNA-seq data.

Table S3 aac.01294-24-s0005.xlsx Oligonucleotides used in the study.

ASM does not own the copyrights to Supplemental Material that may be linked to, or accessed through, an article. The authors have granted ASM a non-exclusive, world-wide license to publish the Supplemental Material files. Please contact the corresponding author directly for reuse.
