# Supplementary figures and images for: Genetic interaction analysis of Candida glabrata transcription factors CST6 and UPC2A in the regulation of respiration and fluconazole susceptibility
Source: Antimicrob Agents Chemother. 2024 Dec 23;69(2):e01294-24. doi: 10.1128/aac.01294-24 (PMC11823675; doi:10.1128/aac.01294-24)

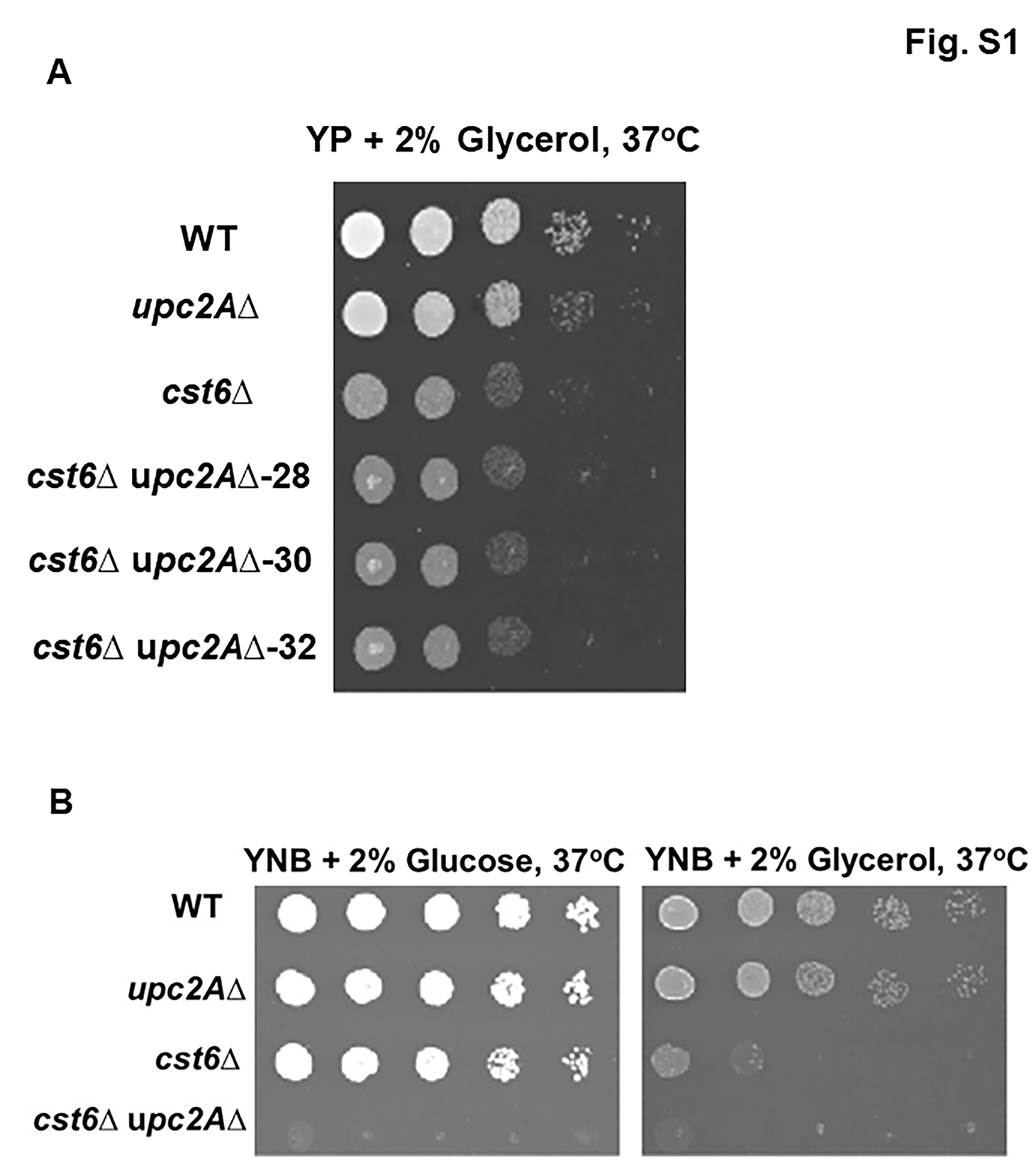

Supplement: Fig. S1 — Additional phenotypes on glycerol-containing medium. [file aac.01294-24-s0001.tif]
