# Supplementary material for: Genetic interaction analysis of Candida glabrata transcription factors CST6 and UPC2A in the regulation of respiration and fluconazole susceptibility
Source: Antimicrob Agents Chemother. 2024 Dec 23;69(2):e01294-24. doi: 10.1128/aac.01294-24 (PMC11823675; doi:10.1128/aac.01294-24)
Supplement: Supplemental legends — Legends for supplemental tables and figure. [file aac.01294-24-s0002.docx]

**Supplementary Materials**

**Supplementary Table S1**. Sterol profile for the *upc2*A∆, *cst6*∆, and *upc2A*∆ *cst6*∆ mutants and BG2 in the presence of fluconazole.

**Supplementary Table S2.** RNA-seq datasets for the *upc2*A∆, *cst6*∆, and *upc2A*∆ *cst6*∆ mutants and BG2 in the presence and absence of fluconazole.

**Supplementary Table S3.** Oligonucleotide primers used in this work.

**Supplementary Figure 1.** **Additional glycerol phenotypic analyses**. **A**. Multiple isolates of *upc2A*∆ *cst6*∆ mutants were plated on YP+ 2% glycerol medium and incubated at 37^o^C for 72hr. All three isolates showed comparable growth on YP+2% glycerol. **B.** The *upc2*A∆, *cst6*∆, and *upc2A*∆ *cst6*∆ mutants were plated on YNB+ 2% glucose and YNB+ 2% glycerol medium and incubated at 37^o^C for 48hr (glucose) and 72hr (glycerol).
